# Social support and partner presence in chronic pain recovery: clarifying the mechanism behind an observed association

**DOI:** 10.1016/j.bjane.2026.844815

**Published:** 2026-08-14

**Authors:** Betul Kozanhan, Mahmut Sami Tutar, Munise Yildiz

**Affiliations:** Department of Anesthesiology and Reanimation, University of Health Sciences, Konya City Hospital, Konya, Turkey

Dear Editor,

We read with interest the prospective longitudinal study by Castro et al.^1^ examining the clinical and psychological evolution of patients with chronic pain undergoing standard multidisciplinary treatment. By integrating pain intensity with quality of life, anxiety, depression, and sleep outcomes, the authors provide valuable real-world insight into the multidimensional nature of chronic pain recovery.

A notable finding was the association between partner presence and clinically meaningful pain reduction (≥ 30%; p = 0.022). In the adjusted analysis, partner presence was associated with clinically meaningful pain reduction.^1^ However, this observational finding should be interpreted cautiously, as the study cannot establish causality or determine the pathway underlying the observed association.

The binary categorization of participants as living with or without a partner cannot capture the quality, nature, or consistency of interpersonal support. Previous literature suggests that social interactions in the context of chronic pain may have heterogeneous associations with pain-related behaviours and adjustment.^2^, ^3^, ^4^ These studies provide possible explanatory frameworks, but they do not establish the mechanisms responsible for the association reported by Castro et al.

We also noted that patients who improved showed a significant increase in SF-36 social functioning scores over the six-month follow-up.^1^ It would be informative to know whether this gain was specifically associated with partner presence or whether it reflected broader social integration. Such an analysis could help clarify whether the observed association was more specifically related to partner presence or reflected wider social support.

In addition, the authors acknowledge the lack of control over medication adherence as a limitation^1^. One untested hypothesis is that partner presence could be associated with practical support in managing complex treatment regimens. However, because treatment adherence was not measured, the available data cannot determine whether adherence contributed to the observed association. Future prospective studies could assess medication adherence, the quality of social support, and partner responses to pain to explore these possible pathways.

Castro et al.^1^ should be commended for drawing attention to a factor that extends beyond conventional biomedical predictors. Future studies incorporating more detailed measures of partner-related and broader social support may help determine whether family-oriented or caregiver-oriented strategies warrant evaluation within multidisciplinary chronic pain programmes, including those delivered in public health settings such as the Brazilian Unified Health System.

## Conflicts of interest

The authors declare no conflicts of interest.
